# Low Enteric Colonization with Multidrug-Resistant Pathogens in Soldiers Returning from Deployments- Experience from the Years 2007–2015

**DOI:** 10.1371/journal.pone.0162129

**Published:** 2016-09-06

**Authors:** Hagen Frickmann, Dorothea Wiemer, Claudia Frey, Ralf Matthias Hagen, Rebecca Hinz, Andreas Podbielski, Thomas Köller, Philipp Warnke

**Affiliations:** 1 Department of Tropical Medicine at the Bernhard Nocht Institute, German Armed Forces Hospital of Hamburg, Hamburg, Germany; 2 Institute for Medical Microbiology, Virology and Hygiene, University Medicine Rostock, Rostock, Germany; 3 Deployment Health Surveillance Capability, NATO Center of Excellence for Military Medicine, Munich, Germany; Seconda Universita degli Studi di Napoli, ITALY

## Abstract

This assessment describes the enteric colonization of German soldiers 8–12 weeks after returning from mostly but not exclusively subtropical or tropical deployment sites with third-generation cephalosporin-resistant Enterobacteriaceae, vancomycin-resistant enterococci (VRE), and methicillin-resistant *Staphylococcus aureus* (MRSA). Between 2007 and 2015, 828 stool samples from returning soldiers were enriched in nonselective broth and incubated on selective agars for Enterobacteriaceae expressing extended-spectrum beta-lactamases (ESBL), VRE and MRSA. Identification and resistance testing of suspicious colonies was performed using MALDI-TOF-MS, VITEK-II and agar diffusion gradient testing (bioMérieux, Marcy-l’Étoile, France). Isolates with suspicion of ESBL were characterized by ESBL/ampC disc-(ABCD)-testing and molecular approaches (PCR, Sanger sequencing). Among the returnees, *E*. *coli* with resistance against third-generation cephalosporins (37 ESBL, 1 ESBL + ampC, 1 uncertain mechanism) were found in 39 instances (4.7%). Associated quinolone resistance was found in 46.2% of these isolates. Beta-lactamases of the bla_CTX-M_ group 1 predominated among the ESBL mechanisms, followed by the bla_CTX-M_ group 9, and bla_SHV_. VRE of vanA-type was isolated from one returnee (0.12%). MRSA was not isolated at all. There was no clear trend regarding the distribution of resistant isolates during the assessment period. Compared with colonization with resistant bacteria described in civilians returning from the tropics, the colonization in returned soldiers is surprisingly low and stable. This finding, together with high colonization rates found in previous screenings on deployment, suggests a loss of colonization during the 8- to 12-week period between returning from the deployments and assessment.

## Introduction

Spread of multidrug-resistant bacteria is a global concern, involving subtropical and tropical war and crisis zones where international armed forces are deployed. Extended-spectrum beta-lactamase (ESBL)-producing Enterobacteriaceae are frequent colonizers in the gastrointestinal tract of civilian returnees from the tropics [1–4]. Contacts with medical infrastructure in central African settings have been described with subsequent colonization rates with ESBL-positive Enterobacteriaceae up to >90% [5]. Antibiotic pressure contributes to the colonization with ESBL-expressing or multidrug-resistant bacteria [1].

Enteric colonization with atypically resistant or multidrug-resistant pathogens does not necessarily mean obligate progression to infections [1], however. Decolonization of the gut in such cases is usually neither possible nor necessary, at least in healthy travelers or returnees. The average colonization time in case of enteric colonization with ESBL-expressing Gram-negative bacteria is estimated to be between a few months and a year, although cases of long-term shedding have been described [6].

In cases of vancomycin-resistant enterococci, however, enteric colonization with resistant bacteria could be shown also to increase the risk of infections due to these pathogens, at least in high-risk populations [7–9]. Both the suppression of the natural flora of the gut and the colonization density were shown to be of prognostic importance for the development of endogenous infections [10, 11]. The increased risk of invasive infections with methicillin-resistant *Staphylococcus aureus* (MRSA) in patients with nasal MRSA colonization can be considered as well established [12].

Studies on enteric colonization of deployed soldiers with atypically resistant and multidrug-resistant pathogens are scarce. Accordingly, only analogous conclusions are possible for military staff. Nevertheless, the hypothesis seems sound that harmless colonization might bear the risk of endogenous infections in physically injured soldiers. Transmission to family members poses a further risk scenario.

As a first step in the assessment of risks due to colonization with resistant bacteria after deployment of personnel, this study assessed the rates of persisting colonization with resistant bacteria in stool samples of soldiers who had returned from deployments 8 to 12 weeks previously to estimate the extent of the problem.

## Materials and Methods

### Study group

The study group consisted of returned soldiers who attended routinely for medical returnee screenings at the Department of Tropical Medicine at the Bernhard Nocht Institute, German Armed Forces Hospital of Hamburg, after military deployments between 2007 and September 2015. Their deployments were mainly but not exclusively in tropical or subtropical settings.

The appointments for the screenings were scheduled between 8 and 12 weeks after the return to Germany. Stool analyses were routinely offered to the returnees as a one-time screening per soldier. A total of 828 returnees provided sufficient quantities of fresh, nonfixed stool and could thus be included in the assessment. The assessed soldiers comprised returnees from Afghanistan (*n* = 172), Argentina (*n* = 2), Bosnia and Herzegovina (*n* = 1), Brazil (*n* = 1), the Central African Republic (*n* = 1), China (*n* = 1), the Democratic Republic of the Congo (*n* = 112), Djibouti (*n* = 16), Ethiopia (*n* = 1), French Guyana (*n* = 5), Gabon (*n* = 6), Ghana (*n* = 9), Indonesia (*n* = 3), Jamaica (*n* = 1), Kosovo (*n* = 42), Lebanon (*n* = 3), Liberia (*n* = 3), Mali (*n* = 43), Malta (*n* = 1), Morocco (*n* = 1), Nigeria (*n* = 13), not-further-defined African destinations (*n* = 3), not-further-specified regions in the Indian Ocean (*n* = 1), Pakistan (*n* = 2), Panama (*n* = 1), Senegal (*n* = 5), Somalia (*n* = 1), South Sudan (*n* = 35), Sudan (*n* = 202), Tanzania (*n* = 7), Thailand (*n* = 3), Uganda (*n* = 41), unknown or multiple deployment settings (*n* = 14), Uzbekistan (*n* = 73), Venezuela (*n* = 1), Vietnam (*n* = 1), and Zimbabwe (*n* = 1).

Returnee-related data associated with the bacterial isolates, i.e. age and sex of the returnees, are not presented for ethical considerations. Participation in the medical returnee screenings was by order. No informed consent was obtained from the returnees regarding the presentation of their personal data in this study. Accordingly, no details regarding the returnees colonized by the strains can be shown.

### Culture screening for resistant bacteria in the stool samples

Cherry-pit-sized volumes from the stool samples provided were used for broth enrichment in thioglycolate broth (Heipha, Eppelheim, Germany) for 16–24 hours at 37°C. Subsequently, 10 μl preincubated broth was cultured on Brilliance ESBL selective agar (Oxoid, Basingstoke, UK) for the selection of third-generation cephalosporin-resistant bacteria. This agar is made for selective growth of ESBL-positive Enterobacteriaceae. An additional 10 μl for each was incubated on Brilliance VRE agar (Oxoid) for the selection of vancomycin-resistant enterococci (VRE) and on CHROMagar MRSA (CHROMagar, Paris, France) for the selection of methicillin-resistant *Staphylococcus aureus* (MRSA).

Agar plates were incubated at 37°C for 40–48 hours. All colonies that looked suspicious for Enterobacteriaceae on Brilliance ESBL selective agar (blue, green, brown colonies) were isolated, while suspected Gram-negative nonfermentative rod-shaped bacteria (i.e., yellow or yellowish-brown or greenish-brown colonies) were discarded. Similarly, only colonies that appeared suspicious for MRSA and VRE were selected for further analysis. Suspected enterococci were of blue or violet color; suspected MRSA were of mauve color.

All suspicious isolates were frozen at −80°C in Microbank tubes (Pro-Lab Diagnostics, Bromborough, UK) until further assessment.

### Identification and phenotypic resistance testing

Identification of isolates was performed with VITEK-II GN-cards (bioMérieux, Marcy-l'Étoile, France) and matrix-assisted laser-desorption-ionization time-of-flight mass spectrometry (MALDI-TOF-MS) using a Shimadzu/Kratos “AXIMA Assurance” MALDI-TOF mass spectrometer (Shimadzu Germany Ltd., Duisburg, Germany). For MALDI-TOF analyses, isolates were prepared using alpha-cyano-4-hydroxycinnamic acid (bioMérieux) as matrix. Spectral fingerprints were analyzed using the Vitek MS-ID IVD-mode database version 3.2.0.-6. (bioMérieux). Automated antibiotic susceptibility testing was performed with VITEK-II AST-N263-cards (bioMérieux). In case of uncertain results, E-testing (bioMérieux) was added. Interpretation of resistance testing results was based on the CLSI 2014 D and the EUCAST guideline (version 4.0, 2014, http://www.eucast.org/fileadmin/src/media/PDFs/EUCAST_files/Breakpoint_tables/Breakpoint_table_v_4.0.pdf, VITEK 2 systems version 06.01). In third-generation cephalosporin-resistant Enterobacteriaceae, the presence of ESBL- or ampC-type resistance was phenotypically confirmed or excluded by the commercial ESBL/ampC disc-based ABCD test kit Mast ID D68C (Mast Diagnostic, Amiens, France) as described by the manufacturer and others [13].

### Genotypic resistance testing

Genotypic resistance typing was performed exactly as described [14]. In short, the approach comprised PCRs with subsequent Sanger sequencing for the *bla*_*TEM*_ and *bla*_*SHV*_ beta-lactamases [15, 16], as well as PCRs for the *bla*_*CTX-M*_ groups I–IV [15, 17]. Of note, group I comprises *bla*_*CTX-M-1*, *-3*, *-10*, *-11*, *-12*, *-15*, *-22*,*-23*, *-28*, *-29*, *-30*_, group II *bla*_*CTX-M-2*, *-4*, *-5*, *-6*, *-7*, *-20*_, group III *bla*_*CTX-M-8*_, and group IV *bla*_*CTX-M-9*, *-13*, *-14*, *-16 to -19*, *-21*, *-27*_ [17, 18].

### Ethical clearance

Ethical clearance for the retrospective assessment of detected multidrug resistant pathogens by the country of deployment was provided by the ethics committee of the University Medicine Rostock (registration number A2015-0077) in line with national and ICH-GCP guidelines.

## Results

### Detected third-generation cephalosporin-resistant Enterobacteriaceae, MRSA, and VRE

Enterobacteriaceae with resistance against third-generation cephalosporins were detected in 39 out of 828 samples analyzed (4.7%). The only species detected was *Escherichia coli* without exemption. The distribution on the regions of deployment that were assessed is shown in Table 1. High percentages of resistant isolates were found predominantly for regions with low numbers of returnees. For a representative selection of countries of deployment, detection rates in returned deployed soldiers, and detection rates in local patients are compared in Table 2. No clear trend for an increase in detected resistant Enterobacteriaceae over the years was demonstrated. The distribution of the detected third-generation cephalosporin-resistant *E*. *coli* ranged from 0% to 18.7% of analyzed samples (Table 3).

**Table 1:** Detection of third-generation cephalosporin-resistant Enterobacteriaceae in returning soldiers.

Country of deployment	Analyzed samples, *n*	Resistant isolates, *n*	Samples with resistant isolates, %
Afghanistan	172	3	1.7
Democratic Republic of the Congo	112	3	2.7
Djibouti	16	1	6.3
Ghana	9	1	11.1
Lebanon	3	1	33.3
Mali	43	3	7.0
Nigeria	13	1	7.7
Not-further-defined African destinations	3	1	33.3
South Sudan	35	7	20
Sudan	202	9	4.5
Tanzania	7	1	14.3
Thailand	3	1	33.3
Uganda	41	3	7.3
Unknown or multiple deployment settings	14	1	7.1
Uzbekistan	73	3	4.1

No third-generation cephalosporin-resistant Enterobacteriaceae were observed in returnees from Argentina, Bosnia and Herzegovina, Brazil, the Central African Republic, China, Ethiopia, French Guyana, Gabon, Indonesia, Jamaica, Kosovo, Liberia, Malta, Morocco, not-further-specified regions in the Indian Ocean, Pakistan, Panama, Senegal, Somalia, Venezuela, Vietnam, and Zimbabwe.

**Table 2:** Detection of enteric colonization with third-generation cephalosporin-resistant Enterobacteriaceae in returned soldiers and previously reported detection rates in selected African and Asian countries (in alphabetic order).

Country	Resistant isolates in returned soldiers, *n* (%)	Percentage of resistant isolates within all analyzed Enterobacteriaceae in studies in the named countries (years of analysis)	References from previous studies
Central African Republic	0/1 (0)	12% of infections (2004–2006)	[19]
China	0/1 (0)	41% colonization (2011–2012) and 38–69% of infections (2011)	[20, 21]
Ethiopia	0/1 (0)	33% of infections (2003–2004)	[22]
Gabon	0/6 (0)	45% colonization (2012) and 15% of infections (2009–2012)	[5, 23]
Ghana	1/9 (11.1)	49% of infections (2011–2012)	[24]
Indonesia	0/3 (0)	2% colonization (2001–2002) and 36% of infections (2005)	[25, 26]
Morocco	0/1 (0)	43% colonization (2012) and 8% of infections (2010–2011)	[27, 28]
Nigeria	1/13 (7.7)	37% of infections (2013)	[29]
Pakistan	0/2 (0)	60% of infections (2009)	[30]
Senegal	0/5 (0)	4% of infections (2004–2006)	[31]
Tanzania	1/7 (14.3)	79% of infections (2011–2012)	[32]
Thailand	1/3 (33.3)	12% of infections (2004–2010)	[33]
Uganda	3/41 (7.3)	79% of infections (2011–2012)	[34]
Vietnam	0/1 (0)	40–49% of infections (2009–2011)	[35]

Interpretation has to be performed with care due to the very low numbers of assessed returned soldiers. A trend to very low colonization rates in the returnees is nevertheless detectable.

**Table 3:** Detection of third-generation cephalosporin-resistant Enterobacteriaceae per year and yearly percentage of respective detections.

Year	Assessed samples, *n*	Resistant isolates, *n*	Samples with resistant isolates, %
2007^a^	405^a^	8	2.0
2008	49	1	2.0
2009	67	3	4.5
2010	47	4	8.5
2011	38	3	7.9
2012	34	0	0
2013	68	4	5.9
2014	75	14	18.7
2015	45	2	4.4

^a^There was a high number of analyses in 2007 because the submission of samples was supervised by the field doctors in the peripheral barracks. In the following years this supervision did not take place.

No MRSA was detected and there was only one VRE isolate (0.1%). The VRE isolate, an *Enterococcus faecalis* strain, was isolated from a returnee from Afghanistan in 2007.

### Phenotypic resistance characteristics of the isolated resistant bacteria

ABCD testing of the 39 third-generation cephalosporin-resistant *E*. *coli* strains demonstrated 37 strains with ESBL-type resistance, 1 strain with a combined ESBL-/ampC-type resistance, and 1 strain with a noninterpretable result pattern. Concomitant cotrimoxazole resistance was frequent, with 29/39 affected strains (74.4%). Resistance against both ciprofloxacin and levofloxacin was observed in 16/39 strains (41.0%); 2/39 additional strains (5.1%) tested intermediate sensitive for ciprofloxacin but sensitive for levofloxacin. Gentamycin resistance was observed in 10/39 isolates (25.6%), and nitrofurantoin resistance in 2/39 (5.1%). Carbapenems, fosfomycin, and tigecycline were effective against all assessed *E*. *coli* strains.

Of note, the single VRE isolate showed high-level resistance against streptomycin but not against gentamycin. Phenotypic E-testing suggested a *vanA*-resistance type (minimum inhibitory concentration (MIC) for vancomycin: 256 μg/ml; MIC for teicoplanin: 32 μg/ml).

### Genotypic analysis of third-generation cephalosporin-resistant *E*. *coli* strains

PCR detected *bla*_*TEM*_- and *bla*_*SHV*_-type beta-lactamases in 8 and 2 stains, respectively. Sequence analysis of the *bla*_*TEM*_-type beta-lactamases showed non-ESBL-associated *bla*_*TEM-1*_ genes in all instances. The affected returnees came from deployments in South Sudan (*n* = 3), the Democratic Republic of the Congo (*n* = 2/3), Afghanistan (*n* = 1/3), Sudan (*n* = 1/9), and Uzbekistan (*n* = 1/3). Sequence analysis of the *bla*_*SHV*_-type beta-lactamases showed *bla*_*SHV-2a*_ in a returnee from Uganda (1/3) and *bla*_*SHV-12*_ in a returnee from Djibouti (1/1). Of note, the strain with the *bla*_*SHV-12*_-type beta-lactamase had shown the noninterpretable pattern in ABCD testing.

Beta-lactamases of the *bla*_*CTX-M*_ group 1 were most frequently observed with 26 positive strains, isolated from soldiers who had returned from South Sudan (*n* = 5/7), Sudan (*n* = 4/9), the Democratic Republic of the Congo (*n* = 3/3), Uganda (*n* = 3/3), Uzbekistan (*n* = 3/3), Afghanistan (*n* = 1/3), Ghana (*n* = 1/1), Lebanon (*n* = 1/1), Mali (*n* = 1/3), Nigeria (*n* = 1/1), not-further-defined African destinations (*n* = 1/1), Tanzania (*n* = 1/1), and unknown or multiple deployment settings (*n* = 1/1). Beta-lactamases of the *bla*_*CTX-M*_ group IV were observed in 6 instances, comprising returnees from Mali (*n* = 2/3), South Sudan (*n* = 2/7), Sudan (*n* = 1/9), and Thailand (*n* = 1/1).

The PCR protocols applied failed to identify the genetic resistance mechanisms in 6 strains from Sudan (*n* = 4/9), and Afghanistan (*n* = 2/3). Each of the 6 strains had been phenotypically characterized as ESBL-positive by ABCD testing.

## Discussion

Influx of resistant pathogens due to soldiers returning from tropical deployments poses a potential public health issue. Close household contacts have been identified as a major source for the spread and as a reservoir for long-term persistence and distribution of resistant bacteria outside of the hospital environment [36].

In Germany, such colonization of soldiers with resistant bacteria is known from the International Security Assistance Force (ISAF) mission in Afghanistan [37, 38]. Further, high colonization rates with ESBL-positive Enterobacteriaceae have been demonstrated in European soldiers with diarrhea during the European Union Training Mission (EUTM) in Western African Mali [14, 39]. Such screening results are not surprising but simply reflect the known phenomenon of increased colonization with ESBL-positive bacteria in civilian returnees from the tropics [1–4]. Resistance surveillance at the respective sites of deployment can contribute to a specific risk assessment.

However, our assessment of enteric colonization with resistant pathogens in German soldiers returning from tropical and nontropical deployments at the Department of Tropical Medicine at the Bernhard Nocht Institute, German Armed Forces Hospital of Hamburg, suggests only low to very moderate colonization of the gut at 8–12 weeks after returning home. MRSA strains were absent and VRE virtually absent, while the colonization rates with ESBL-positive Enterobacteriaceae resembled the situation in Germany or were only slightly increased for most deployment settings. Accordingly, a broth enrichment protocol was used to increase sensitivity as has been suggested [40]. Broth enrichment increases the yield of resistant bacteria after swabbing: e.g., by a factor of 2 for ESBL-expressing bacteria in upper respiratory tract samples [41]. The selective agar media used were reported to show good sensitivity and to be rather likely to lack specificity for the VRE as shown in a previous study [42]. In detail, sensitivity of 94.9–97.9% and specificity of 95.7–100% have been reported for Brilliance ESBL selective agar [43, 44]. This agar also detects *Klebsiella* spp. and other Enterobacteriaceae with resistance against third-generation cephalosporins, so the identification of resistant *Escherichia coli* only is not due methodical features. The reported sensitivity and specificity of CHROMagar MRSA are 95.4–100% and 95–100%, respectively [45–47]. The sensitivity and specificity of Brilliance VRE agar are reported to be 95% and 87.1%, respectively [42]. For *vanB-*type VRE, a comparable sensitivity of 94% has been described [48].

Considering that soldiers are ordered to come to the Department of Tropical Medicine for returnee assessments if they have been particularly exposed to the often suboptimal local hygiene conditions on deployment, the low colonization rates after return in comparison with the high rates at the deployment sites suggest a rapid drop after the end of deployment.

This hypothesis is in line with previously published data from the civilian setting, indicating a high acquisition rate but mostly short durations of carriage of multidrug-resistant Enterobacteriaceae in travelers returning from tropical areas [49]. The postulated risk of household-associated spread [36] is therefore highest shortly after return. Stricter adherence to standard hygiene precautions in the weeks after return, possibly combined with screening efforts, therefore seems advisable.

Nevertheless, some soldiers’ guts remained colonized even 8–12 weeks after the end of deployment, allowing for potential intermediate-term to long-term spread of resistant bacteria. The displacement of resistant bacteria acquired from deployments by susceptible flora of the gut remains a stochastic process that can to some extent be predicted at population level [6] but not so far for the individual patient. At present, reliably efficient methods for the eradication of resistant bacteria from the gastrointestinal tract are also not available [50].

Colonization with resistant bacteria makes the formulation of antibiotic therapy difficult in cases of severe endogenous infection in military forces, both during subtropical and tropical deployments [14, 37–39] and—to a considerably lesser degree, as shown here—also after deployment. The deployment history of soldiers should therefore be included in consideration of antimicrobial therapeutic approaches.

In the deployment setting, microbiological routine diagnostic equipment is usually not available in small medical units. Therefore, it is advisable to avoid antibiotic therapy in case of questionable indications—in order not to select resistant strains—but to use broad-spectrum or even combined antibiotic therapy in case of emergency indications to override expected resistance in line with available surveillance data. If the appropriate microbiological laboratory equipment is available in medical field camps to permit cultural growth and resistance testing, resistance-guided antibiotic therapy should be preferred for the treatment of severe infections on deployment.

Although a substantial number of stool samples from returned soldiers were analyzed, the study is limited by the small numbers of returnees from various deployment settings, the shifting of the deployment sites over time, and the retrospective design of the study. Further, pre-deployment samples prior to the missions and samples taken directly after deployments were not available, preventing patient-specific serial comparisons. Future studies should address these issues.

Considering that the analysis was performed 8–12 weeks after the return of the soldiers from deployment, acquisition of the resistant colonizing isolates back in Germany cannot be completely excluded. In the same way, one cannot exclude persistent colonization that already existed prior to the deployments in individual cases, because no pre-deployment samples were taken. Phylogenetic typing, e.g. based on multi-locus sequence typing (MLST) [51] or next-generation sequencing (NGS) [52], would also not have been suitable to identify the place of acquisition of the bacteria for the following reasons. First of all, no MLST or NGS databases exist so far that cover the study sites of deployment in a comprehensive way to allow for a confirmation or exclusion of origin of the bacteria from the respective sites. Even if clonal complexes that have been described in individual papers to be prevalent in certain geographic regions had been identified, this would not have definitely excluded transmission after the soldiers’ return home. Soldiers typically work in contact with other soldiers who are also on various international deployments, so nosocomial transmission of strains of regionally unusual clonal complexes can occur back in Germany as well. Typing to exclude nosocomial transmission [51, 52], on the other hand, was not useful because the described isolation events did not occur in a temporally associated manner that would make nosocomial transmission etiologically plausible. Because the additional information from typing approaches such as MLST or NGS would have been marginal, these assessments were not performed. The general finding that the enteric colonization of returning German soldiers with resistant bacteria after deployment including subtropical and tropical deployment settings is low at assessment time points 8–12 weeks after deployment remains unaffected by this.

Given the finding that persistence of resistant pathogens as enteric colonizers can still be detected 8–12 weeks after the end of deployments, screening of military returnees for long-term shedding of resistant bacteria seems advisable. Although reliable eradication from the gut cannot be offered, results will allow for individualized consultation on hygiene precautions regarding household transmission [36], in particular if severely ill or even immune-compromised patients at risk of acquiring potentially life-threatening infections [7–9] occupy the same accommodation as the affected soldier.
